# How a picture becomes a word: individual differences in the development of language-mediated visual search

**DOI:** 10.1186/s41235-020-00268-9

**Published:** 2021-01-04

**Authors:** Sarah Chabal, Sayuri Hayakawa, Viorica Marian

**Affiliations:** Department of Communication Sciences and Disorders, Northwestern University, 2240 North Campus Drive, Evanston, IL 60208 USA

## Abstract

Over the course of our lifetimes, we accumulate extensive experience associating the things that we see with the words we have learned to describe them. As a result, adults engaged in a visual search task will often look at items with labels that share phonological features with the target object, demonstrating that language can become activated even in non-linguistic contexts. This highly interactive cognitive system is the culmination of our linguistic and visual experiences—and yet, our understanding of how the relationship between language and vision develops remains limited. The present study explores the developmental trajectory of language-mediated visual search by examining whether children can be distracted by linguistic competitors during a non-linguistic visual search task. Though less robust compared to what has been previously observed with adults, we find evidence of phonological competition in children as young as 8 years old. Furthermore, the extent of language activation is predicted by individual differences in linguistic, visual, and domain-general cognitive abilities, with the greatest phonological competition observed among children with strong language abilities combined with weaker visual memory and inhibitory control. We propose that linguistic expertise is fundamental to the development of language-mediated visual search, but that the rate and degree of automatic language activation depends on interactions among a broader network of cognitive abilities.

## Significance statement

Whether it is an immunologist looking through a microscope or a lost child looking for a familiar landmark, adults and children alike routinely rely on their ability to identify specific objects in complex visual scenes. Existing research has demonstrated that adults’ visual search patterns can be biased by irrelevant linguistic information. Here, we show that a similar phenomenon occurs in children as young as eight years old and that children’s visual search patterns are impacted by individual differences in not only linguistic expertise, but also visual memory and domain-general inhibitory control. In particular, our findings suggest that the extent of linguistic influence over visual processing depends on both the likelihood of initial language activation (e.g., the relative efficiency of phonological vs. visual processing), as well as the ability to suppress language when it impedes, rather than facilitates, search performance. These results have implications for identifying characteristics and contexts that are most likely to elicit linguistic bias. Furthermore, we show that the well-established visual world paradigm can be used to track children’s language development as they progress from the acquisition of declarative linguistic knowledge (e.g., vocabulary) to procedural linguistic processing (e.g., automatic language activation). We conclude that understanding the role of development and individual differences in language-vision interaction may contribute to how well we can predict whether language will impact visual search, and provide clinicians and researchers with tools for assessing the automaticity of children’s language processing.

Goal-directed visual search serves multiple functions in our daily lives—it can help a doctor locate a malignant tumor or help a lost child find their way home. Though the stakes and complexity of the task can vary across individuals and contexts, two features that are likely to span most search tasks are that they are grounded in our early developmental experiences, and that they can be influenced by our linguistic knowledge. Here, we explore both elements by examining the emergence of language-mediated visual search in children.

Chabal and Marian (2015a) demonstrated that when adults engage in *non-linguistic* visual search for an object (e.g., a *belt*), they often make visual fixations towards other items that have similar sounding labels (e.g., a *bell*), even if no language input has been provided. As we learn the words that enable us to describe the things we see, linguistic and visual representations can become so closely associated that language is automatically activated when we process visual scenes. Though researchers have continued to uncover contextual and individual variables that moderate language-vision interactions for adults (Görges et al. 2013; Marian et al. 2014; Meyer et al. 2007; Walenchok et al. 2016; Zelinksy and Murphy 2000), less is known about how this interactivity emerges during the course of development.

The automatic retrieval of labels associated with visual objects is likely contingent on extensive experience associating linguistic and visual representations (Huettig and McQueen 2007; Huettig et al. 2012). Because children have less language experience than adults, their visual search processes may be less impacted by language (Sekerina and Brooks 2007; Swingley et al. 1999). For example, Sekerina and Brooks (2007) found that when children identified a visual target in response to a spoken word, they experienced less phonological competition compared to adults (see also Snedeker and Trueswell 2004 for evidence that children’s language processing is less influenced by visual cues). In fact, the trajectory of linguistic influence appears to be incremental, with greater language activation developing with greater linguistic expertise.

The impact of language on how children engage in visual search is additionally likely to be moderated by individual traits. For instance, language-vision interactivity may depend on the relative development of visual vs. phonological memory (Hayes and Birnbaum 1980; Hitch et al. 1989). Children who are more adept at recalling visual information may prioritize visual features in search displays over the linguistic characteristics associated with them. Moreover, domain-general functions such as cognitive control may impact language activation during visual search, as studies with adults suggest (Blumenfeld and Marian 2011; Hayakawa et al. 2020). Research with children has additionally shown a positive association between visual search performance and executive function, including inhibitory control (Datin-Dorrière et al. 2020), working memory (Ólafsdóttir et al. 2019), and IQ (Cornish et al. 2008), as well as between eye-witness memory and inhibitory control (Roberts and Powell 2005). Using independent measures of language aptitude, visual ability, and inhibitory control, the present study was designed to assess whether children activate language when engaged in non-linguistic visual search and to identify individual differences that augment the developmental course of linguistic influence over visual processing.

## Methods

### Participants

Twenty-four native English speakers (12 male) aged 8–12 years old (*M* = 10.04; SD = 1.38) participated in the experiment. All participants reported normal vision, no history of language or learning disabilities, and no history of hearing impairments.

### Design and materials

The experiment was based on a non-linguistic search task, in which volunteers saw a visual object and then searched for that object in a subsequent display. The 2 × 2 repeated-measures design contained picture type (competitor, control) and target condition (target-present, target-absent) as within-subject variables. Individual difference scores on assessments of language ability, visual memory, and inhibitory control were also considered as independent variables in order to determine how language competition may be impacted by children’s cognitive and linguistic development.

The dependent variables of interest were the duration and proportion of visual fixations to linguistic competitor and control items. To the extent that children activate the labels of the visual objects, we would expect that linguistic overlap with the target would draw attention toward the competitor object, resulting in more visual fixations to the competitor relative to the control. Both target-present and target-absent trials were included in the experiment so that we could explore whether linguistic competition is contingent upon the simultaneous activation of objects' labels or whether competition can be observed in a sequential manner. On the one hand, we may expect greater competition when the target is present, as this would provide opportunities to activate the target label during both the preview and search stage. Assuming that the target label is indeed activated during the preview stage, however, participants may experience increased competition when the target is absent, as this could encourage greater consideration of non-target objects and, possibly, greater reliance on the target label to confirm its absence.

Thirty stimulus sets were constructed based on items previously shown to elicit phonological competition in adults (Chabal and Marian 2015a). On target-present trials, displays included a target object (e.g., a *drum*) and a phonological competitor whose English label shared an average of 2.53 (SD = 0.63) initial phonemes with the target (e.g., a *dress*). Displays additionally included a control (e.g., a *carrot*) and a filler (e.g., a *frog*) that did not share initial phonological overlap with any other item in the set. On target-absent trials, the target was replaced with an additional filler item. Object positions were counterbalanced across trials, with competitors and controls always adjacent to the target and fillers diagonal to the target. Trial-order was pseudorandomized and counterbalanced across subjects. All objects were depicted by black and white drawings chosen from the International Picture Naming Project database (Bates et al. 2003) or were independently normed using Amazon Mechanical Turk. Labels of each stimulus type were matched on word frequency, orthographic/phonological neighborhood density, concreteness, familiarity, imageability, and age of acquisition (*p*s > .05).

**Fig. 1:**
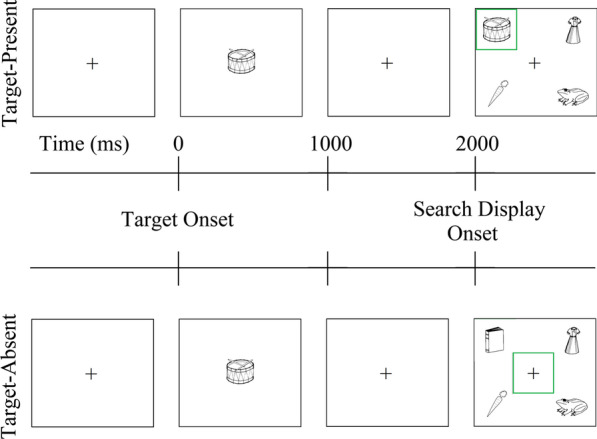
Example target-present (top) and target-absent (bottom) trials. Participants were shown a preview of a target (e.g., *drum*) followed by a search display, which included either a target (e.g., *drum*, in the target-present condition) or a filler (e.g., *book*, in the target-absent condition), as well as a phonological competitor (e.g., *dress*) and a control (e.g., *carrot*) adjacent to the target position, and a filler (e.g., *frog*) diagonal to the target position. Participants were instructed to click on the target if it was present and the central fixation cross if it was absent

Participants completed 60 critical trials (30 target-present, 30 target-absent) and 90 filler trials. On each trial, the participant was presented with the target picture for 1000 ms, followed by a fixation cross, which was replaced by the four-object search display after 1000 ms. The search display remained on the screen until the participant provided a response. Each trial was preceded by an inter-stimulus interval of 1500 ms. Participants were instructed to click on the target as quickly as possible if it was present and to click on the center fixation cross if it was absent (Fig. 1).

### Procedure

Prior to the experiment, informed consent was obtained from participants’ legal guardians; children provided verbal and written assent. Participants were familiarized with the desk-mounted eye-tracker (EyeLink1000 Version 1.5.2, SR Research Ltd.), which had a sampling rate of 1000 Hz. Calibration was obtained using a nine-point calibration and validation procedure with drift correction. Following the search task, participants verbally provided names for each of the target and competitor items seen throughout the experiment. Prior to analyses, incorrectly named or unnamed images were discarded individually for each participant (17.5% of trials).

Participants then completed the NIH Toolbox Cognition Battery (Weintraub et al. 2013), from which Picture Vocabulary, Picture Memory, and Flanker scores were *z*-score transformed and used as individual difference measures of language ability, visual memory, and inhibitory control, respectively.

### Data analysis

The *duration* and *proportion* of fixations to competitor and control items were analyzed with separate linear mixed effects regressions using the lme4 package (Bates et al. 2014) in R (R Core Team 2016). Both models included fixed effects of target condition (target-absent: − 0.5 vs. target-present: + 0.5), competition (control: − 0.5 vs. competitor object: + 0.5), each individual difference measure (language ability, visual memory, inhibitory control), and all interactions. Participants’ *z*-score transformed age (in months) was included as a covariate. The models additionally included maximal random effect structures, with random intercepts for subject and trial, by-subject random slopes for target condition and competition, and by-trial random slopes for competition and each of the individual difference measures. Trials that were responded to incorrectly (2.36% of trials) or trials in which the log-transformed response time was two standard deviations above or below the mean (4.31% of trials) were excluded from the fixation analyses. Analyses of accuracy and response time can be found in “Appendix.”

## Results

**Fig. 2:**
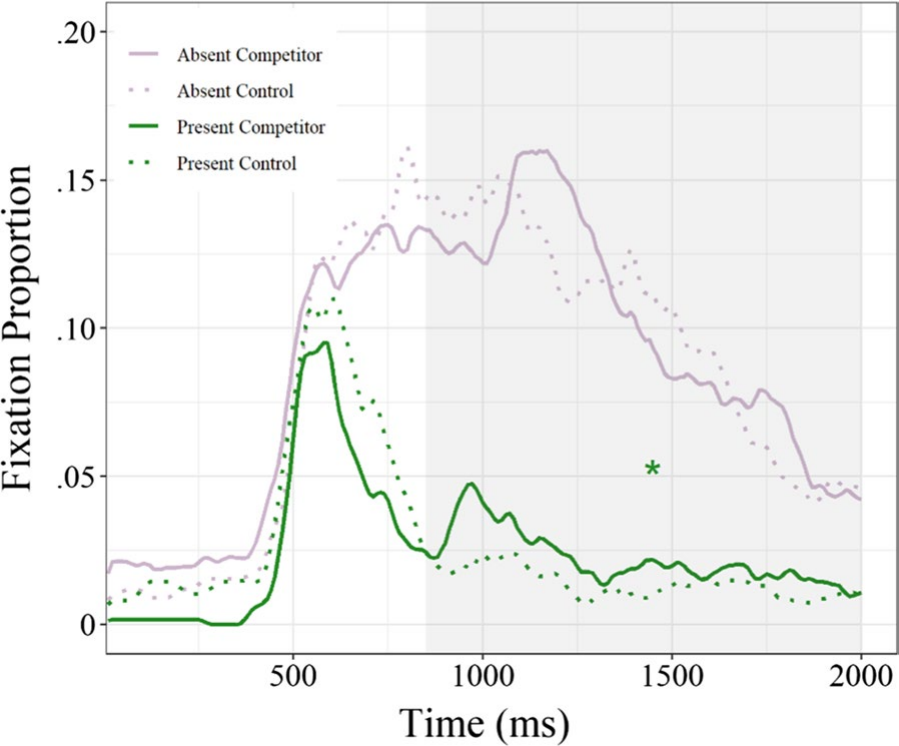
Timecourse of visual fixations to competitor (solid) and control objects (dotted) in the target-absent (purple) and target-present (green) conditions. Competitor and control fixations were longer and more frequent when the target was absent. When the target was present, competitor fixations were longer and more frequent than control fixations from 850 ms following presentation of the competitor (shaded area)

There were significant main effects of target condition for the duration (*Estimate* = − 18.38, SE = 4.59, *t*(182.47) = − 4.00, *p* < .0001) and proportion (*Estimate* = − 0.09, SE = 0.01, *t*(20.51) = − 16.24, *p* < .0001) of fixations, with longer and more frequent looks to both competitors and controls when the target was absent (see Fig. 2). The main effects of competition were not significant for the duration (*Estimate* = 4.89, SE = 4.94, *t*(127.43) = 0.99, *p* = .323) or proportion (*Estimate* = − 0.002, SE = 0.006, *t*(30.22) = − 0.33, *p* = .740) of fixations, suggesting that language activation during visual processing may be less robust for children compared to adults. Visual inspection of fixations over time, however, indicates that some competition may have emerged in the middle of the time window, which was confirmed when the analyses were restricted to visual fixations occurring at least 850 ms following presentation of the competitor. Specifically, we found that there was a significant effect of competition for both the duration (*Estimate* = 35.83, SE = 15.55, *t*(83.67) = 2.30, *p* = .024) and proportion (*Estimate* = 0.01*, *SE = 0.01, *t*(99.96) = 2.11, *p* = .035) of fixations during this time window when the target was present, but not when it was absent (*p* > .05 for both duration and proportion).

The primary models including all time points additionally revealed a significant four-way interaction between target condition, competition, visual memory, and inhibitory control for the duration (*Estimate* = − 27.56, SE = 11.38, *t*(1070.07) = − 2.42, *p* = .016) and proportion (*Estimate* = − 0.02, SE = 0.01, *t*(1784) = − 2.33, *p* = .032) of fixations. There were significant interactions between target condition, language ability, and visual memory (*Estimate* = 0.02, SE = 0.01, *t*(16.56) = 2.36, *p* = .031), between target condition, visual memory, and inhibitory control (*Estimate* = 0.02, SE = 0.01, *t*(14.89) = 2.60, *p* = .020), and between target condition, language ability, visual memory, and inhibitory control (*Estimate* = − 0.02, SE = 0.01, *t*(17.84) = − 2.33, *p* = .032) for the proportion model. No other effects (including age) were significant (*p*s > .05).

To clarify the nature of the relationships between competition, target condition, and individual differences, we computed the average competition effects (competitor—control fixations) for each participant, which were then analyzed separately for each target condition using linear models. Separate models were then constructed for the duration and proportion of (relative) competitor fixations, with fixed effects of language ability, visual memory, and inhibitory control, plus all interactions. No random effects were included, as each participant contributed a single index of competition per model.^1^

### Target-Absent

**Fig. 3:**
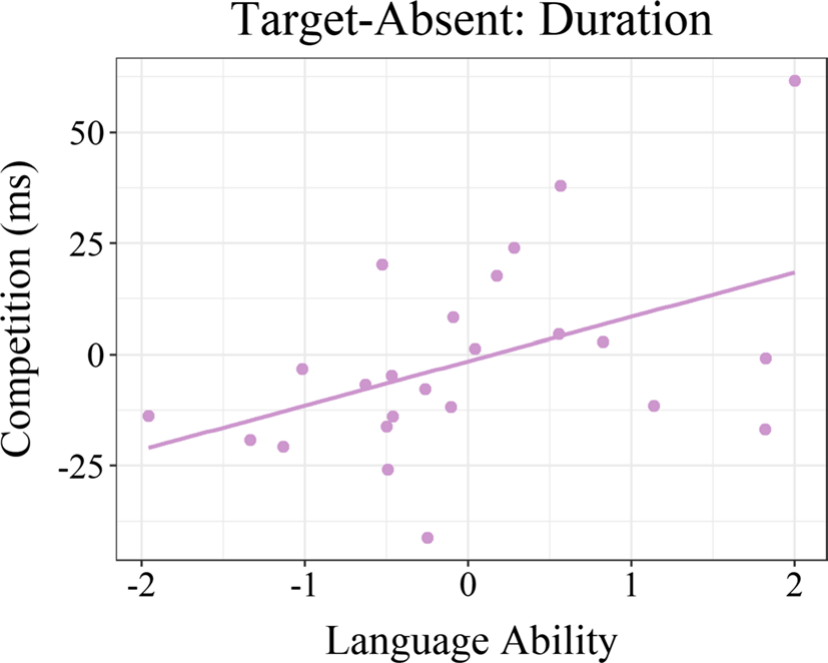
Relationship between language ability (*z-*score) and the relative duration of competitor fixations (competitor—control) in milliseconds. Higher scores were associated with significantly greater phonological competition

For target-absent trials, there was a significant main effect of language ability on the relative duration of competitor fixations, with increased competition for children with higher language ability scores (*Estimate* = 12.21, SE = 3.71, *t*(16) = 3.29, *p* = .005; see Fig. 3). In other words, compared to children with weaker language abilities, children with stronger language skills were more likely to look at the competitor objects for longer than the control objects.

**Fig. 4:**
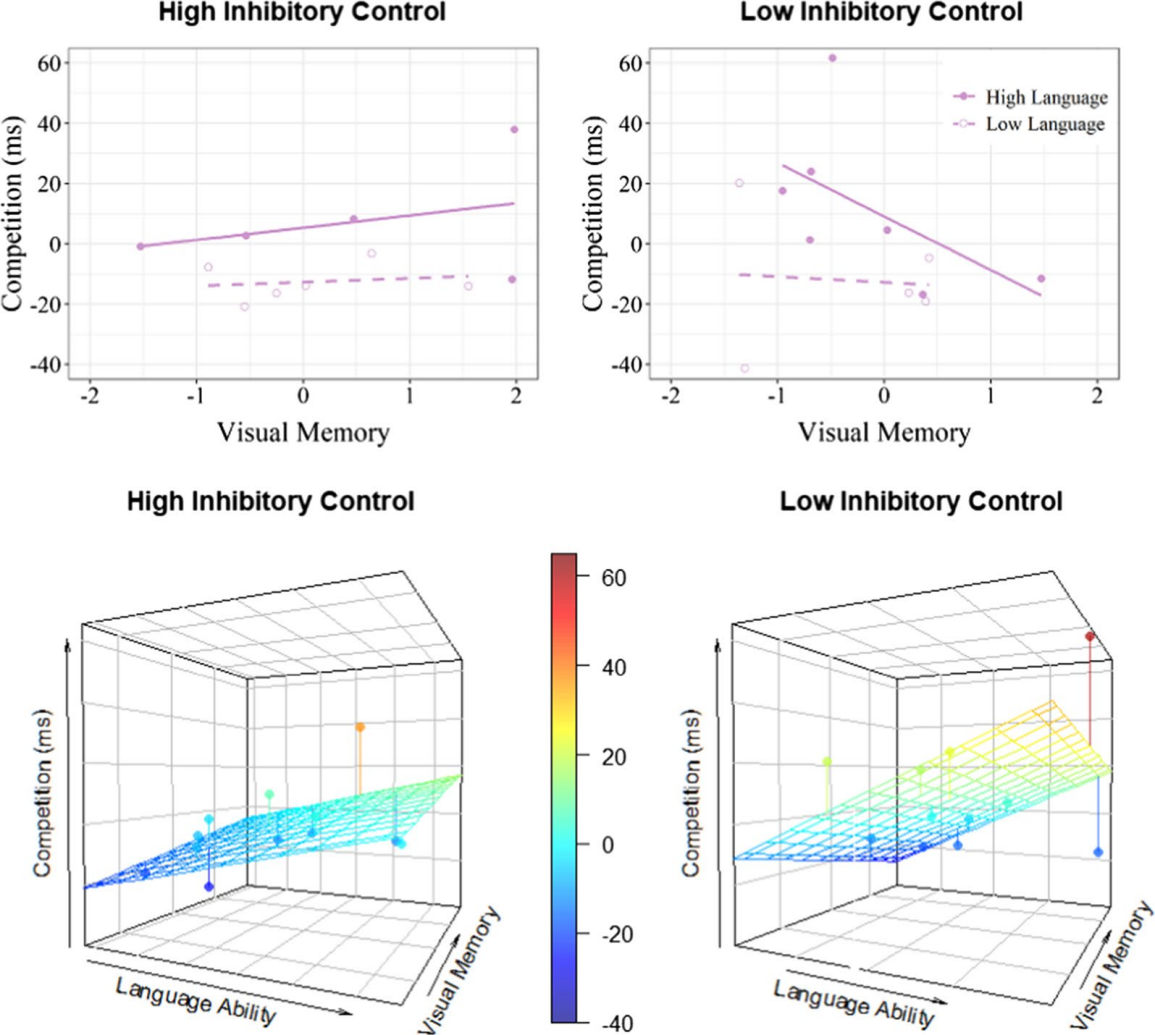
**a** Observed relationship between visual memory (*z*-score) and the relative duration of competitor fixations (competitor—control) for children with high (left) or low (right) inhibitory control and high (solid) or low (dashed) language ability. Positive values indicate longer looks to competitor than control objects. **b** Observed (dots) and predicted (grid) relative competition by visual memory and language ability for children with high (left) or low (right) inhibitory control. Redder shades indicate greater competition. Children with better language ability (i.e., larger vocabularies) generally experienced greater phonological competition, especially when combined with lower visual memory and lower inhibitory control

Additionally, a significant three-way interaction emerged between language ability, inhibitory control, and visual memory (*Estimate* = 15.22, SE = 5.72, *t* = 2.66, *p* = .017). To follow-up on this interaction, we examined the effect of visual memory on competition by first dividing participants into high and low language ability groups based on the median language score; these groups were then further divided into high and low inhibitory control (IC) groups based on median IC scores within each language ability group (High Language/High IC, High Language/Low IC, Low Language/High IC, and Low Language/Low IC). Though we did not find significant effects of visual memory within groups, there was a notable pattern where lower visual memory scores were associated with greater phonological competition for individuals in the High Language/Low IC group (*Estimate* = − 26.12, SE = 23.31, *t*(4) = − 1.12, *p* = .325). The effects of visual memory for the remaining three groups, on the other hand, were relatively more modest (*p*s > .70; see Fig. 4). In other words, better language ability was generally associated with greater phonological competition, but this may have been especially the case for individuals with low inhibitory control and low visual memory. No significant effects were found for the relative proportion of competitor fixations (*p*s > .05).

### Target-Present

**Fig. 5:**
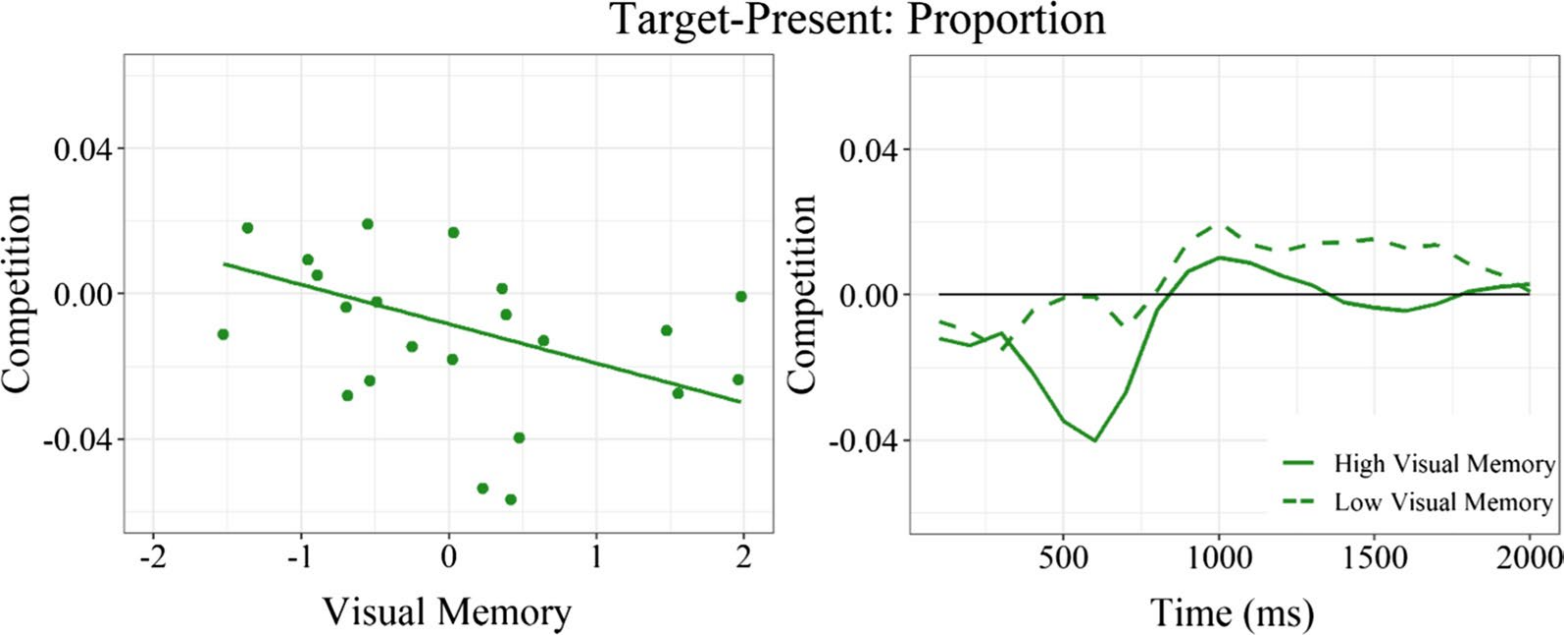
Relative proportion of competitor fixations (competitor—control) by *z*-scored visual memory (left) and over time for children with high (solid) and low (dashed) visual memory (right). Lower visual memory was associated with significantly greater phonological competition. Positive values indicate more looks to competitor than control objects

For target-present trials, no effects approached significance for the relative duration of competitor fixations (*ps* > .05). There was, however, a significant main effect of visual memory on the relative proportion of competitor fixations (*Estimate* = − 0.02, SE = 0.01, *t* = − 2.87, *p* = .011). Comparable to the trend observed for target-absent trials, greater phonological competition was associated with lower visual memory scores (see Fig. 5). In other words, compared to children with stronger visual memory, children with weaker visual memory were more likely to look at competitor objects more often than control objects.

**Fig. 6:**
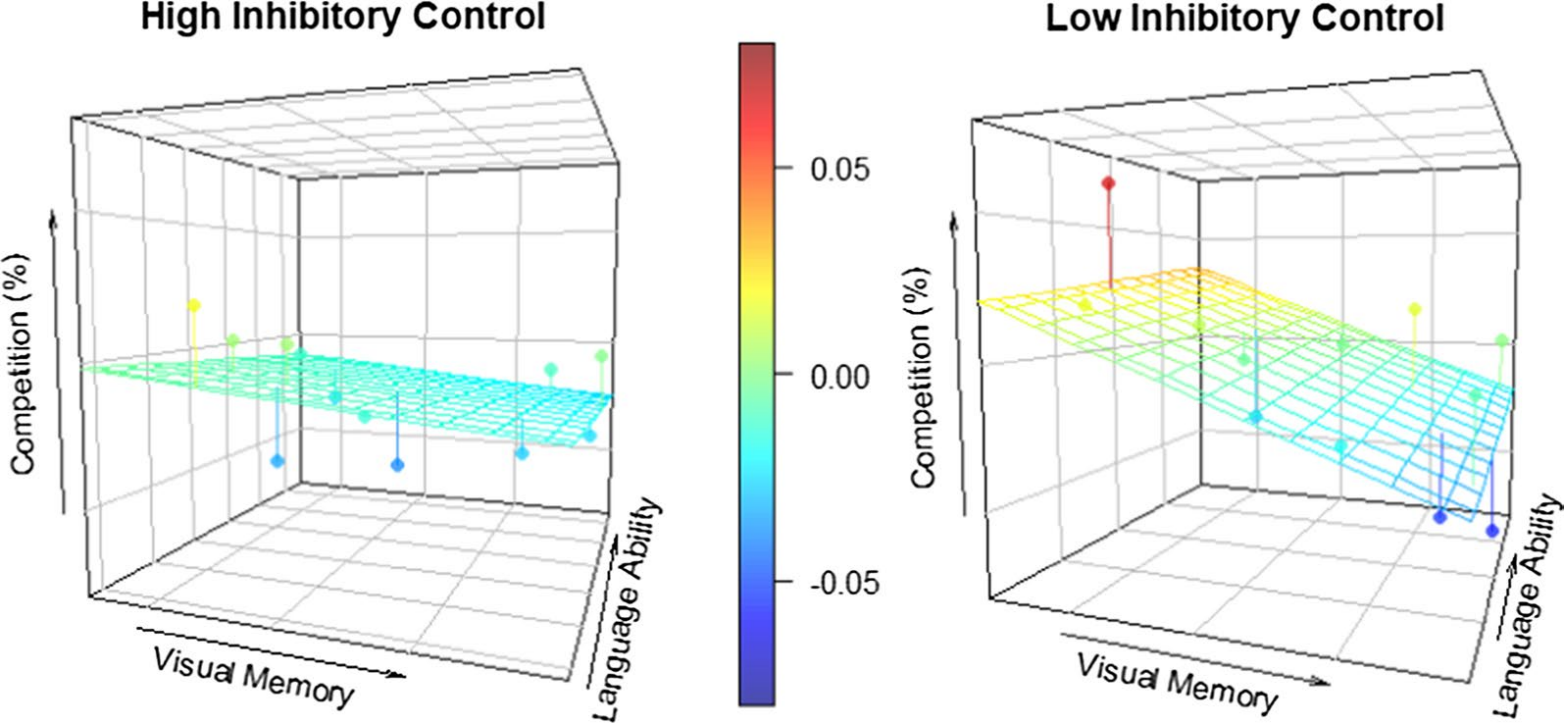
Observed (dots) and predicted (grid) phonological competition (competitor—control fixation proportion) by visual memory and language ability for children with high (left) or low (right) inhibitory control. Redder shades indicate greater competition. Lower visual memory was associated with greater competition, especially for children with lower inhibitory control

Also similar to target-absent trials, visual inspection suggests that competition may be greatest among children with low inhibitory control in addition to low visual memory (see Fig. 6). Though the effect of visual memory did not interact with either inhibitory control or language ability (both *ps* > .05), the simple effect of visual memory was significant for children with low (*Estimate* = − 0.27, SE = 0.01, *t* = − 2.48, *p* = .038), but not high (*Estimate* = − 0.01, SE = 0.01, *t* = − 1.34, *p* = .216), inhibitory control.

## Discussion

When conducting a visual search task, adults’ visual fixations are impacted by the linguistic features of objects within the visual display (Chabal and Marian 2015a). In the present study, we demonstrate that, although linguistically based competition between visual objects is not as robust among typically developing children, evidence of competition can be found from approximately 850 ms following the simultaneous presentation of objects whose labels share phonological features. Furthermore, we find that individual differences in language ability (i.e., vocabulary size) mediate how children process visual scenes. Specifically, language-based competition (i.e., longer fixations to competitors relative to controls) was observed in approximately one-third of participants, and all but one of these children belonged to the high language ability group based on a median split.

The observed pattern of results is consistent with models of language-vision interaction positing that visually based linguistic activation emerges from extensive experience associating linguistic and visual features of a given object (see Huettig and McQueen 2007). Just as we found that phonological competition was more pronounced among children with greater linguistic expertise, Chabal and Marian (2015a) found larger competitor effects when adult English–Spanish bilinguals encountered competition in their dominant language (English). Similarly, though phonological competition during visual search has been reliably observed when visual objects are associated with real words (as in Chabal and Marian 2015a), the effects of competition are more tenuous when participants are trained to associate visual stimuli with novel words (Zelinsky and Murphy 2000). Our finding that children do not activate language as readily as adults during non-linguistic visual tasks confirms the hypothesis that visually based language activation is modulated by how much experience an individual has associating linguistic labels with visual referents. Despite developmental differences in the prevalence of phonological competition, some degree of language-mediated visual search can already be observed in children as young as 8 years old, with the extent of competition related to individuals’ language knowledge.

Not only do our data support that language ability mediates linguistic competition during visual search, but they also provide preliminary evidence that the effects of linguistic aptitude may be moderated by individual differences in visual memory and executive processing during the course of development. Specifically, we observed a trend on target-absent trials for phonological competition to be most likely to emerge among children who have high language ability but low visual memory and inhibitory control. Moreover, we found that on target-present trials, lower visual memory increased phonological competition independently of language ability. The design of the present study, in which children were tasked with identifying the exact same visual object that was shown to them mere moments before, may have been particularly well-suited to capturing these individual differences attributed to visual memory. Only children who were unable to form a usable visual template of the target item (i.e., those with lower visual memory abilities) relied on the activation of that object’s name to help them remember their goal on the subsequent search display. Therefore, we might expect that the influence of language during visual search would be observed in a larger subset of children if there were a greater benefit of accessing linguistic and/or semantic knowledge, such as if the target was explicitly cued with a linguistic label or if the search display included a different exemplar of the target category. Therefore, the present findings are likely to represent a conservative estimate of phonological competition.

Though phonological competition has been observed among adults regardless of whether language serves a purpose for the task (Chabal and Marian 2015a; Chabal et al. 2020), holding an object’s label in mind can facilitate visual memory and search (Logie et al. 2016; Lupyan and Swingley 2012). In fact, language can often be used to bootstrap performance for a variety of cognitive functions, such as by highlighting subtle distinctions between categories (Lupyan 2006) and providing “perceptually-simple correlates to an otherwise perceptually-complex task” (Lupyan 2006, p. 195). In the context of the current study, children who had difficulty encoding the perceptual features of the target may have relied on linguistic categories to create a meaningful search template. As individuals are confronted with increasingly complex challenges over the course of development, it may be that the more consistent language activation observed in adults stems, in part, from the implicit or explicit acquisition of linguistically based strategies. If so, it is possible that early difficulties in other domains may, perhaps paradoxically, accelerate the development of more sophisticated forms of cognitive processing (see Mayberry 2002).

The way that children’s inhibitory control seems to moderate linguistic competition during visual search is also consistent with findings in adult populations (Blumenfeld and Marian 2011; Hayakawa et al. 2020). For instance, executive control regions (e.g., anterior cingulate, superior frontal gyrus) are activated during a visual search task when monolingual adults resolve within-language competition (Marian et al. 2014), as well as when bilinguals resolve between-language competition (Marian et al. 2017).

It is of note, however, that the effect of inhibitory control on phonological competition was largely restricted to individuals with stronger linguistic abilities. It is therefore likely that the role of inhibitory control in the early stages of language-vision interactions is secondary to the acquisition of linguistic expertise and would be expected to have a more ubiquitous impact at later stages of development. This hypothesis is not unprecedented, as research suggests that linguistic experience can have a direct influence on the development of cognitive control (Blumenfeld and Marian 2011; Gangopadhyay et al. 2019; Chabal and Marian 2015b) and that individual differences in executive function predict language outcomes (Bartolotti et al. 2017; Blumenfeld et al. 2016)—in large part due to variability in how often and how well individuals manage linguistic interference.

Lastly, the present findings confirm that the visual world paradigm (VWP), which has been used extensively with adult populations, may provide a useful means for capturing individual differences in children’s language processing that extend beyond explicit tests of linguistic knowledge. Though we found that the degree of linguistic competition during visual search was associated with overall vocabulary size, the present study only included trials for which the target and competitor labels were known. In other words, declarative knowledge of words associated with objects in a visual scene does not, in itself, guarantee that they will impact visual search—rather, visual fixations to linguistic competitors are contingent on automatic language activation and the development of proceduralized forms of language processing. We propose that this critical aspect of children’s language competence (that is often overlooked in favor of explicit measures of declarative knowledge) can be quantified using the methods outlined in the present investigation, with potential implications for clinicians, researchers, and educators. Future extensions may therefore focus on replicating the present findings with larger populations of linguistically and socially diverse children, both to confirm the reliability of the observed effects, as well as to validate the procedure for use in applied contexts.

In sum, the present findings demonstrate that the emergence of language-mediated visual search is modulated by individual differences in language ability, visual memory, and inhibitory control. The developmental process whereby language becomes intertwined with the visual world is thus likely to extend beyond linguistic and visual processing to involve a broad network of bidirectional relationships among multiple cognitive abilities.

## Figures and Tables

**Fig. 7:**
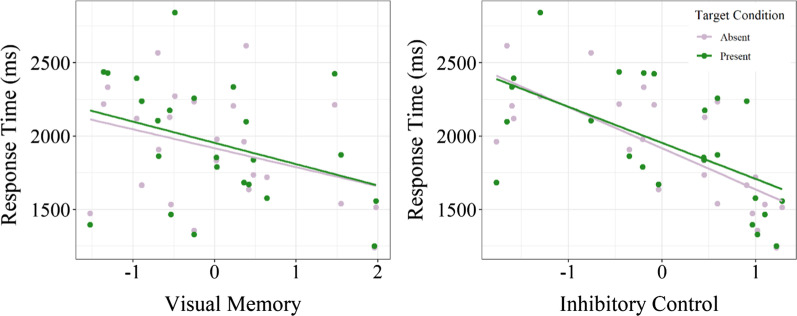
Response times for target-absent (purple) and target-present (green) trials in relation to *z*-score transformed individual differences for visual memory (left) and inhibitory control (right). Higher scores for both measures were associated with faster response times

## Data Availability

The datasets analyzed during the current study are available from the corresponding author on reasonable request.

## Appendix: Accuracy and response time analyses

### Data analysis

Accuracy and response time (RT) were analyzed with separate mixed effects regressions using the lme4 package (Bates et al. 2014) in R (R Core Team 2016). Both models included fixed effects of target condition (target-absent: − 0.5 vs. target-present: + 0.5), each individual difference measure (language ability, visual memory, inhibitory control), and all interactions. They additionally included random intercepts for subject and stimulus set, a by-subject random slope for target condition and by-set random slopes for target condition and each of the individual difference measures. Log-transformed RT was analyzed using a linear mixed effects model and accuracy was analyzed using a generalized mixed effect model.

### Results

Accuracy on target-absent trials (*M* = 99.33%, SD = 8.19) was numerically, but not significantly, higher than on target-present trials (*M* = 95.79%, SD = 20.10; *Estimate* = − 5.99, SE = 3.71, *z* = − 1.62, *p* = .106). Most errors on target-present trials (93.75%) resulted from mistakenly indicating that the target was not present. RT was marginally faster with better visual memory (*Estimate* = − 0.07, SE = 0.03, *t*(13.96) =  − 3.29, *p* = .056) and significantly faster with better inhibitory control (*Estimate* = − 0.11, SE = 0.03, *t*(14.67) =  − 3.29, *p* = .005; see Fig. 7). Language ability had no significant effect on RT (*p* > .05). In other words, though the more nuanced eye-tracking measures revealed that children with stronger language skills experienced greater competition, this did not translate to slower response times overall. Age was not associated with accuracy or RT, and no other effects were significant for either model (*ps* > .05).

## Footnotes

1 Including age as an additional fixed effect did not notably change the pattern of results in follow-up analyses.
